# Thyroid Hormones, Thyromimetics and Their Metabolites in the Treatment of Liver Disease

**DOI:** 10.3389/fendo.2018.00382

**Published:** 2018-07-10

**Authors:** Marta A. Kowalik, Amedeo Columbano, Andrea Perra

**Affiliations:** Unit of Oncology and Molecular Pathology, Department of Biomedical Sciences, University of Cagliari, Cagliari, Italy

**Keywords:** T3, T2, thyromimetics, thyroid hormone receptor, NASH, hepatocellular carcinoma, regenerative medicine, liver cell proliferation

## Abstract

The signaling pathways activated by thyroid hormone receptors (THR) are of fundamental importance for organogenesis, growth and differentiation, and significantly influence energy metabolism, lipid utilization and glucose homeostasis. Pharmacological control of these pathways would likely impact the treatment of several human diseases characterized by altered metabolism, growth or differentiation. Not surprisingly, biomedical research has been trying for the past decades to pharmacologically target the 3,5,3′-triiodothyronine (T3)/THR axis. *In vitro* and *in vivo* studies have provided evidence of the potential utility of the activation of the T3-dependent pathways in metabolic syndrome, non-alcoholic fatty liver disease (NAFLD), and in the treatment of hepatocellular carcinoma (HCC). Unfortunately, supra-physiological doses of the THR agonist T3 cause severe thyrotoxicosis thus hampering its therapeutic use. However, the observation that most of the desired beneficial effects of T3 are mediated by the activation of the beta isoform of THR (THRβ) in metabolically active organs has led to the synthesis of a number of THRβ-selective thyromimetics. Among these drugs, GC-1, GC-24, KB141, KB2115, and MB07344 displayed a promising therapeutic strategy for liver diseases. However, although these drugs exhibited encouraging results when tested in the treatment of experimentally-induced obesity, dyslipidemia, and HCC, significant adverse effects limited their use in clinical trials. More recently, evidence has been provided that some metabolites of thyroid hormones (TH), mono and diiodothyronines, could also play a role in the treatment of liver disease. These molecules, for a long time considered inactive byproducts of the metabolism of thyroid hormones, have now been proposed to be able to modulate and control lipid and cell energy metabolism. In this review, we will summarize the current knowledge regarding T3, its metabolites and analogs with reference to their possible clinical application in the treatment of liver disease. In particular, we will focus our attention on NAFLD, non-alcoholic steatohepatitis (NASH) and HCC. In addition, the possible therapeutic use of mono- and diiodothyronines in metabolic and/or neoplastic liver disease will be discussed.

## Thyroid hormone T3 and liver disease

Thyroid hormones (THs), in particular 3,5,3'-triiodo-L-thyronine (T3), have long been recognized to regulate multiple physiological processes, including fetal development, cell growth, homeostasis, as well as carbohydrate, lipid, protein, and mineral metabolism (1). Through the years, numerous effects of the thyroid hormone excess (a result of administration of exogenous TH or excessive activity of the thyroid gland) on lipid metabolism have been reported, such as increased metabolic rate, weight loss, lipolysis, lowering of serum cholesterol levels; in addition, the improvement in myocardial contractility has been described (2). Although the physiological actions of TH affect almost every organ, liver is one of the most important targets of TH. In fact, alteration of cellular TH signaling has been reported to cause liver-associated diseases, such as non-alcoholic fatty liver disease (NAFLD) and hepatocellular carcinoma (HCC), major health problems worldwide (3). NAFLD, which includes two pathologically distinct conditions associated to different prognoses—non-alcoholic fatty liver (NAFL) and non-alcoholic steatohepatitis (NASH)—is the most common liver disorder in Western countries, affecting 17–46% of adults (4–6). In particular, the acronym NASH, coined by Ludwig et al. (7), describes a liver disease that histologically mimics alcoholic hepatitis but occurs in individuals whose alcohol consumption is nil or negligible. The diagnosis of NASH provides important prognostic information and indicates an increased risk of fibrosis progression, cirrhosis and HCC (6). Despite its high prevalence and potentially serious consequences, so far no effective drug treatment for NASH has been provided. In this regard, exogenous T3 administration showed encouraging results in lowering hepatic fat content in various experimental models of NAFLD. A nutritional model often utilized in NAFLD studies is the choline-devoid methionine-deficient (CMD) diet that results in massive hepatic accumulation of triglycerides and liver injury with close pathological and biochemical similarities to human NASH (8, 9). In this model, T3 completely prevented the development of hepatic steatosis by increasing fatty acid mitochondrial and peroxisomal β-oxidation and decreasing the expression of liver-type fatty acid-binding protein (L-FABP) (10). Most important, T3 also promoted regression of pre-existing fatty change. The disappearance of hepatic triglycerides was associated with a strong decrease of lipid peroxidation, cyclooxygenase-2 (COX-2) expression, phospho-signal transducer and activator of transcription 3 (phospho-STAT3) and phospho-stress-activated protein kinase/c-Jun NH2-terminal kinase (phospho-SAPK/JNK) levels (10), usually activated in inflammatory processes (11). The following analysis of the effects of T3 on lipid homeostasis *in vivo*, its effects on plasma free fatty acid (FFA) levels, hepatic gene expression, and mitochondrial respiration rates, evaluated in Sprague-Dawley rats, demonstrated that, in the liver, T3 reduced the mRNA levels of sterol regulatory element binding protein-1c (SREBP-1c) and apolipoprotein C3 (ApoC3) and increased apolipoprotein A1 (ApoA1), peroxisome proliferator-activated receptor γ coactivator-1α (PGC-1α) levels (12). Moreover, mRNA levels of carnitine palmitoyltransferase-1 (CPT-1), which catalyzes an essential step for the mitochondrial uptake of long-chain fatty acids and their subsequent β-oxidation in the mitochondria (13), tended to be increased following treatment with T3. Further, a recent report revealed that T3 decreases hepatic fat content in the livers of rats fed high fat diet (HFD); this effect was associated with a concomitant increase of CPT-1 protein levels (14). On the other hand, T3 did not lower triacylglycerol levels in ob/ob mice; this was likely due to the relatively low dose used in this study as chronic T3 administration at higher doses was poorly tolerated in these animals. Moreover, a pronounced increase in heart weight was found in T3-treated animals (12). In the attempt to clarify the mechanisms underlying the anti-steatotic capacity of T3, some studies on T3-regulated cellular pathways that lead to the generation of free fatty acids from stored lipid droplets in the liver, suggested that TH stimulation of fatty acid β-oxidation is also coupled with induction of hepatic autophagy to deliver fatty acids to mitochondria (15). With regard to human data, a recent report, presenting an example of a patient with NASH complicated by Graves' disease, indicated that hyperthyroidism may improve the pathological condition of NASH (16). Besides obesity, NAFLD is also associated with another manifestation of the metabolic syndrome, the insulin resistance (17). Although the cellular mechanism underlying this association is still not fully elucidated, it is believed that by products of the *de novo* lipogenesis, that is up-regulated in NAFLD, favor the hepatic insulin resistance (18). Several investigations with animal models of NAFLD clearly indicate that reduction in fatty liver results in an amelioration of insulin resistance. These experimental evidences suggest that TH, or their analogs, may elicit an insulin sensitizing and antidiabetic effect by reducing hepatic fat accumulation.

Although experimental evidences indicate that T3 actions on hepatic metabolism are mediated exclusively by peripheral actions, either through membrane or nuclear receptors, a growing interest for the study of possible central mechanisms has recently emerged. In particular, Alvarez-Crespo and colleagues have demonstrated that chronic central infusion of T3 significantly affects thermogenesis and adipose tissue activity (19). Although the effects on hepatic metabolism was not investigated, it is possible that similar central effects of T3 on liver also exist.

As previously mentioned, the diagnosis of NASH is associated with an increased risk of HCC (6). It follows that reversal of fat accumulation/liver damage may reduce HCC development. In view of the positive results of T3 on NASH, the effects of T3 administration have been also investigated in experimental models of HCC. Significantly, this tumor type represents the second cause of global cancer-related deaths (20, 21). Since the efficacy of traditional pharmacological therapies and of new developed drugs, such as the multikinase inhibitors sorafenib and regorafenib, and their ability to produce a significant survival benefit remains still questionable (22), there is an urgent need to develop novel therapies for HCC. Several studies collectively indicate that disruption of TH signaling is involved in the development of HCC (23–25). Interestingly, *in vivo* studies showed that a short treatment with T3 accelerated the regression of chemically induced hepatic pre-neoplastic lesions in rats subjected to the Resistant-Hepatocyte (R-H) model of hepatocarcinogenesis (26). This experimental model consists of hepatocyte initiation through a single dose of the carcinogen diethylnitrosamine (DENA) and promotion of initiated hepatocytes through a short-term exposure to 2-acetylaminofluorene (2-AAF) combined with 2/3 partial hepatectomy (PH) (27). Using this protocol, the authors observed that only 50% of rats exposed to repeated cycles of T3 developed HCC, with no sign of lung metastasis (26). An anti-preneoplastic effect of T3 was also observed in the CMD nutritional model (28). T3 has been also recognized as a strong inducer of liver cell proliferation in rats and mice (29–31), which performs its hepatomitogenic effect in the absence of activation of transcription factors, such as AP-1, NF-κB or STAT3, associated with an increased expression of *c*- *fos, c-jun*, or *c-myc* proto-oncogenes and an increase in the mRNA and protein levels of cyclin D1 (31). The potent hepatomitogenic effect of T3 was demonstrated not only for intact liver, but also during the regenerative response in rodents after 70% partial or 90% subtotal hepatectomy (32–35). These results strongly indicate the possible therapeutic use of T3 in conditions characterized by an impaired regenerative ability (such as aged livers) or when a rapid growth stimulation of the liver is required.

## Deleterious effects of T3

The above mentioned beneficial effects of T3 treatment on liver disease are unfortunately counterbalanced by harmful effects on the heart, muscle and bone, as increased T3 levels are known to induce thyrotoxicosis marked by tachycardia, arrhythmia, muscle wasting, and also reduced bone mineralization and alteration of central nervous system development (36, 37). Since all these actions hamper the potential therapeutic use of T3, successive attempts were aimed at identifying THs derivatives, with the purpose of reaping the benefits from T3 treatment by separating the therapeutic actions of T3 from its deleterious effects. The demonstration that the antitumoral effect of T3 is associated with an activation of the THR-mediated pathway in hepatocytes clearly indicates that the effect is mediated by a peripheral action of T3.

## Thyromimetics, THRβ ligands, and liver-targeted TH agonists

TH exert their physiological effects by binding to specific nuclear receptors (38–41), the thyroid hormone receptors (THR) α and β, that exhibit conspicuous differences in expression pattern. The β isoform is the major THR expressed in the liver, whereas the α isoform is particularly abundant in the heart (38, 39). The observation that most of the desired therapeutic effects of T3 in the liver are mediated by the activation of the isoform β of THRs and that THR activation in extrahepatic tissues leads to altered cardiovascular function and thyroid hormone axis (THA) suppression, has led to the synthesis of a number of THRβ- and organ-selective (liver-selective) thyromimetics. Although isoform selective agonists of the THRs have been developed primarily for the treatment of hypercholesterolemia, as an alternative strategy to 3-hydroxy-3-methyl-glutaryl-coenzyme A reductase (HMG-CoA reductase) inhibitors (known as statins) (42, 43), their use has been also proposed for the treatment of several metabolic disorders, including NAFLD and even HCC. In the last 20 years, an improved understanding of THR structure and function has led to the development of several potent thyromimetics, including GC-1, GC-24, KB141, KB2115, and MB07344, the active form of the prodrug MB07811(43–45).

GC-1 (3,5-dimethyl-4 (4′-hydroxy-3′- isopropylbenzyl)-phenoxy) acetic acid, also known as Sobetirome), the first THRβ agonist to be generated and a scaffold compound for the development of other TH derivatives, has been synthetized in 1998. The structural changes presented by GC-1, with respect to the natural hormone T3, allowed GC-1 to demonstrate comparable affinity for THRβ1 to that of T3 and a 10-fold lower affinity to THRα1 compared with T3 (46). Successively, GC-1 has been described to combine both, organ- and THRβ1-selectivity, which enhanced hepatic targeting (47). Importantly, in the same study, GC-1 did not elicit harmful effects on heart weight, heart rate and mRNAs coding for proteins related to cardiac contraction, such as myosin heavy chain α (MHCα), MHCβ and sarcoplasmic reticulum calcium adenosine triphosphatase (Serca2) (48). With regard to the liver disease, Perra *et al*. (10), employing the CMD diet, demonstrated that, the therapeutic effects of T3 on NAFLD were shared by GC-1, which prevented the development of hepatic steatosis and promoted regression to pre-existing fat accumulation in the same model. The reduction in hepatic triglycerides was accompanied by a concomitant decrease of lipoperoxidation (10). GC-1 ameliorated hepatic steatosis also in other animal experimental models, such as ob/ob mice and Western diet-fed LDLR^−/−^ mice (49). Furthermore, GC-1 treatment prevented the development of hepatic steatosis in rats placed on high fat diet; however, at the same time, it caused hyperglycemia and insulin resistance (50). With regard to tumorigenesis, a 14-day treatment with GC-1 caused an almost complete disappearance of hepatic pre-neoplastic lesions in rats subjected to the R-H model of hepatocarcinogenesis (28). Similar to what observed with T3, following GC-1 treatment, the induction of a preneoplastic hepatocyte differentiation program preceded the loss of the preneoplastic lesions, as confirmed by a progressive loss of fetal markers such as the placental form of glutathione S-transferase (GST-P) and gamma glutamyl transpeptidase (GGT) and reacquisition of glucose 6-phosphatase (G6Pase) and adenosine triphosphatase (ATPase), two proteins expressed in normal differentiated liver. An anti-preneoplastic effect of GC-1 was also reported in DENA and CMD diet fed rats (28). These results, together with the finding that the isoform β of THRs is profoundly down-regulated in rat preneoplastic lesions and in rat and human HCC (51), strongly suggest that reactivation of the TH-THR axis may have a strong impact on HCC progression. Another evidence highlightening GC-1 as an attractive candidate in HCC therapy stems also from a recent study (52). By exploiting a model of HCC driven by the co-expression of S45Y-β-catenin and hMet using SBTT and HTVI (53), the authors found that GC-1 exerted a notable anti-tumoral effect by decreasing tumor burden without affecting β-catenin and its downstream targets, therefore, demonstrating its safety for use in chronic liver diseases (52).

Columbano and co-workers demonstrated that GC-1 mimics also the effect of T3 as a powerful inducer of hepatocyte proliferation in rats and mice (54, 55). Furthermore, pre-treatment of mice with GC-1, prior to partial hepatectomy, caused a significant increase in hepatocyte proliferation, suggesting that GC-1 administration confers a regenerative advantage following 2/3 PH (56). In the light of these results, GC-1 might result beneficial also in the area of hepatic regenerative medicine.

The nineties witnessed also the development of GC-1 derivative, GC-24 (57). Although an important improvement in key metabolic parameters, such as plasma triglycerides, body fat, glucose tolerance, and insulin sensitivity were achieved with GC-24 treatment without affecting cardiac weight in rats fed a high-fat diet, GC-24 treatment did not manage to restore hypercholesterolemia, increased hepatic cholesterol content, elevated non-esterified free fatty acids (NEFA) and IL6 levels (58).

Although another THRβ1 agonist, KB141 [3,5-dichloro-4-(4-hydroxy-3-isopropylphenoxy) phenylacetic acid], was successively identified yielding promising results in preclinical animal models, such as significant cholesterol, lipoprotein (a), and body-weight reduction with minimal cardiac side effects, it has not been pursued for human use (45, 59). Moreover, in contrast to GC-1, it was observed that it did not accumulate preferentially in the liver (60, 61).

More recently, novel THR-selective analogs have been reported in the literature, namely KB2115 (Eprotirome, 3-((3,5-dibromo-4-(4-hydroxy-3-(1-methylethyl)-phenoxy)-phenyl)-amino-3-oxopropanoic acid) and MB07811 (2*R*,4*S*)-4-(3-chlorophenyl)-2-[(3,5-dimethyl-4-(4′-hydroxy-3′-isopropylbenzyl)phenoxy)methyl]-2-oxido-[1,3,2]-dioxaphosphonane). KB2115, a compound containing two bromides, was shown to have minimal uptake in non-hepatic tissues, to be more liver-specific than GC-1 and do not provoke deleterious effects on the heart (62, 63). In order to determine whether KB2115 would be a more effective therapy for NAFLD and hepatic insulin resistance compared with GC-1, the effects of KB2115 treatment were evaluated in high-fat diet-fed male Sprague-Dawley rats. Similar to what observed with GC-1, KB2115 treatment effectively prevented the development of hepatic steatosis in this animal model. Unlike GC-1 administration, KB2115 treatment did not lead to fasting hyperglycemia, although it still resulted in fasting hyperinsulinemia. Moreover, in contrast to GC-1, KB2115 treatment did not cause a significant change in glycerol turnover, or in plasma non-esterified fatty acid concentration (50). Similar to GC-1, KB2115 was able to reduce the burden of hepatic steatosis also in ob/ob mice, markedly reducing hepatic triglyceride levels (49). Furthermore, KB2115 shares the hepatomitogenic activity of T3 and GC-1 in the absence of significant signs of liver toxicity, indicating its utility for regenerative therapies in liver transplantation or other surgical settings (64). At present, no data regarding the effect of KB2115 on HCC development/progression are available.

Relevant data confirming the possible importance of THR agonists in the treatment of liver disease have been also obtained with the prodrug MB07811, which undergoes first-pass hepatic extraction and its cleavage generates the negatively charged THR agonist (3,5-dimethyl-4-(4′-hydroxy-3′-isopropylbenzyl)phenoxy)methylphosphonic acid (known as MB07344) (65). Comparison of the effects of MB07811 with T3 and a non-liver-targeted TR agonist, KB-141, on the expression of THR agonist-responsive genes in the liver and extrahepatic tissues, demonstrated enhanced liver targeting for MB07811 with increased cardiac sparing. Importantly, limiting THR activation to the liver resulted in the reduction not only in cholesterol but also in both hepatic and plasma triglyceride levels in Diet-Induced Obese (DIO) mice (65). Successively, using several experimental models of NAFLD, such as Zucker diabetic fatty (ZDF) rats, *ob/ob* mice, diet-induced obese (DIO) mice, Cable and colleagues (12) demonstrated that MB07811 markedly reduced hepatic steatosis, plasma FFA and triglycerides. Importantly, no sign of liver fibrosis or other histological liver damage, transaminase increase were observed after treatment with MB07811. No increase in heart weight was also reported. This antisteatotic activity of MB07811 was likely attributable to an increased metabolic rate in liver and an increased rate of mitochondrial β-oxidation, in particular of CPT-1 levels.

Concerning human clinical trials, GC-1, KB2115 and MB07811 were reported to reduce significantly low-density lipoprotein (LDL)-cholesterol and triglycerides levels (45, 66, 67). However, despite these encouraging results, no compounds that reached human clinical trials developed into therapeutics so far. KB2115 was discontinued after long-term dosing in dogs, demonstrating adverse effects on cartilage (68). Similarly, no phase II trials for GC-1 and MB07344 have been performed (43).

## Recent advances in THRβ ligands

More recently, a new, liver-directed with low extra-hepatic permeability, THR-β agonist has been developed, the 2-[3,5-Dichloro-4-(5-isopropyl-6-oxo-1,6-dihydropyridazin-3-yloxy)phenyl]-3,5-dioxo-2,3,4,5-tetrahydro[1,2,4]triazine-6-carbonitrile, also known as MGL-3196 (69). MGL-3196 resulted to be 28-fold selective for THRβ over THRα in an *in vitro* functional coactivator recruitment assay. Notably, compared with other compounds, no impact on the central thyroid axis and no cardiac effects in a rat heart model were also reported for MGL-3196. In mice, this drug was able to induce a reduction in cholesterol and in liver size, which is secondary to reduction of liver triglycerides. Apparently, no effect on bone mineral density (BMD) or heart or kidney size was detected in MGL-3196-treated animals (69). By contrast, while T3 treated animals demonstrated cholesterol lowering, there was no effect on liver size. In humans, 60% reduction in triglycerides was observed for doses ranging from 50 to 200 mg, in the absence of drug-related adverse events (70). Using HFD-fed mice, it was shown that at human equivalent doses, MGL-3196 reversed and prevented progression of lipid, inflammatory and fibrotic markers of NASH in this model without the adverse effects of T3. Importantly, beneficial effects of MGL-3196 have been also observed in NASH patients, as this THRβ agonist significantly decreased hepatic fat in patients with NASH compared with placebo group. At the moment, MGL-3196 is in Phase 2 for treatment of NASH. As discussed for T3, the modulation of cell metabolism by synthetic THR agonists could be mediated by both central and peripheral actions, with a prevalence of the latter. On the other hand, the proliferative and antineoplastic actions of some agonists appear to be exclusively mediated by their binding to the target cell, as also demonstrated by their *in vitro* activity.

## TH metabolites: 3,5-diiodo-l-thyronine (T2)

Although TH actions were thought to be classically mediated by T3 and 3,5,3′,5′-tetraiodo-L-thyronine (thyroxine or T4), over the last decades, another TH-related compound, 3,5-diiodo-L-thyronine (T2), received marked attention as it was demonstrated to be a bioactive compound. T2, similar to T4, T3 and rT3 (reverse T3), is one of four natural iodothyronines with significant, biological activities. Although experimental *in vitro* evidences for the conversion of T3 to T2 are still lacking, indirect evidence indicates that T2 is the product of the deiodination of the outer ring of T3 and it is reported to have 50–1,000 times lower affinity for THR than T3 (71, 72). TH deiodination can be mediated by three selenoenzymes: type 1 deiodinase (D1), type 2 D2, type 3 D3 (73); among these enzymes, D2 (71) results as the most likely candidate involved in T2 formation (72). Literature data published in the past years proposed that both THR- and non-genomic actions may be elicited by T2; in addition, T2 has been suggested to mimic some of the effects of T3 on liver metabolism (74–76). The results and mechanisms regarding the T2 actions have been explored both *in vitro* and *in vivo*. With regard to the *in vitro* models, the effects of T2 administration were evaluated using primary cultures of rat hepatocytes exposed to an oleate/palmitate mixture in order to obtain “fatty hepatocytes,” a condition mimicking fat accumulation induced *in vivo* by high fat diet (HFD) (77). Similar to T3, the addition of T2 to “fatty hepatocytes” resulted in a significant reduction of lipid content and lipid droplet diameter, as well as in the activities of acyl-CoA oxidase (AOX), a rate-limiting enzyme of peroxisomal β-oxidation, and antioxidant enzymes (78). The beneficial anti-steatotic effects of T2 *in vivo* have been described in several models of NASH, with particular attention to animals fed HFD, a model which has the advantage to mimic most features of human fat overload and overnutrition and allows to study obesity and liver related disorders (79). Notably, T2 was able not only to prevent hepatic steatosis (80) but also to reverse pre-existing hepatic fat accumulation (81), similarly to what observed with T3 (10). In detail, the simultaneous co-treatment of T2 to rats receiving a HFD caused a complete disappearance of hepatic steatosis and a significant reduction in the serum triglyceride and cholesterol levels when compared with rats receiving HFD alone. These effects were the consequence of stimulation of hepatic fatty acid oxidation and CPT activity. No change in heart rate during or at the end of the treatment was observed (80). T2 was also able to markedly reverse HFD-induced hepatic steatosis. Regression of fat accumulation was associated to enhanced fatty acid oxidation rate, CPT activity and improved mitochondrial oxidative stress (81). Successive proteomic studies (82) provided an integrated view of the metabolic adaptations occurring after HFD+T2 treatment. In particular, pathways involved in fatty acid and ketone-bodies metabolism, amino acid and nitrogen metabolism, urea cycle, respiratory chain activity and reactive oxygen species (ROS) production were identified (82). Further studies demonstrated that T2 administration prevented HFD-induced insulin resistance (83). They also suggested that T2 does not act via THRβ, but directly *activates* the hepatic nuclear sirtuin 1 (SIRT1) which was identified as a main factor in mediating the effects of T2. This nuclear deacetylase, that modulates lipid metabolism and enhances mitochondrial activity (84), has been already recognized to contribute to the regulation of hepatic gene expression by T3 through a direct interaction with THRβ (85–87). In addition to the induction of oxidative pathways, T2 was reported to up-regulate the levels of apo-lipoprotein B, the major protein component of very low-density lipoprotein (VLDL) (88), indicating that T2 stimulates lipoprotein secretion to reduce the hepatic fat excess (89). Moreover, in the same study, T2 treatment was shown to affect the levels of adipose triglyceride lipase (ATGL), which selectively performs the first and rate-limiting step of triglycerides hydrolysis to generate diacylglycerol and fatty acids, triggering the lipolytic cascade (90). Very recently, using a targeted metabolomics approach, Iannucci and colleagues (14) deepened and compared the effects of T2 and T3 on the early metabolic adaptation in the livers of rats fed HFD. Both T2 and T3 diminished hepatic triglyceride levels, strongly induced autophagy and intra-hepatic acylcarnitine flux, while preventing the generation of sphingolipid-ceramides, which are known to contribute to the oxidative stress in NASH (91). The effects of T3 and T2 on insulin/growth factor signaling effectors such as mitogen-activated protein kinase (MAPK) and phosphatidylinositol 3-kinase (PI3K) were also investigated. Evaluation of phosphorylation of extracellular signal-regulated kinase (ERK) and Protein kinase B (AKT), demonstrated that only T2 was able to rescue the impairment in AKT and MAPK/ERK pathways caused by HFD (14). As recently demonstrated, although both T2 and T3 have the capacity to regulate lipid accumulation in the liver of rats fed a HFD, they act by different molecular mechanisms to achieve inhibition of hepatic lipid accumulation. While the effects exerted by T2 are accompanied by a decreased lipogenesis and an increased fatty acid oxidation, those exerted by T3 are primarily dependent on increased fatty acid oxidation (92). Although T2 reduced hepatic triglyceride content also in diet-induced obese mice, increased heart weights indicated potential cardiac side effects going beyond hepatic thyromimetic actions (93). However, it should be underlined that the dose of T2 chosen for this study was 10-fold higher than those reported in previous studies, in which prevention of hepatic lipid accumulation without undesirable side effects was observed.

No deleterious side effects on the thyroid axis or at the cardiac level, together with an increased basal metabolic rate and decreased body weight, were observed in two euthyroid subjects who underwent T2 administration. Serum levels of FT3, FT4 and TSH resulted unchanged in these patients (94).Recently, a novel functional analog of T2, 3-[4-(7-hydroxy-6-methyl-indan- 4-ylmethyl)-3,5-dimethyl-pyrazol-1-yl]-propionic acid (TRC150094, TRC) has been tested in rats receiving a HFD. A significant increase in mitochondrial fatty acid import and oxidation led to the reduction of the hepatic triglyceride content in these animals (95). TRC induced also a reduction in cholesterol levels. Importantly, no undesirable effects were observed on heart rate or heart weight in HFD+TRC-treated rats. Next, oral administration of TRC150094 to obese Zucker spontaneously hypertensive fatty rats (obese ZSF1) decreased hepatic steatosis by inducing a significant increase in mitochondrial respiration as well as by increasing fatty acids oxidation (96). Since most of the metabolic effects on hepatocytes elicited by T2 are mediated by a direct action on mitochondria and are reproducible *in vitro*, it is likely that they are mediated by peripheral actions. Nonetheless, the possibility that T2 affects hepatic metabolism by a central action cannot be excluded and requires more experimental evidences. No data regarding the potential therapeutic use of T2 for HCC have been reported so far.

It should be underlined that D3 action generates 3,3′,5′-triiodothyronine (reverse T3), from T4 and another form of T2, 3,3′-diiodo-L-thyronine (3,3′-T2) from T3 (72, 97). However, the comparison of the effects of T3, 3,5-T2 and 3,3′-T2 on metabolic parameters and glucose levels in HFD-fed mice revealed that 3,3′-T2 lacks beneficial metabolic effects and even worsens metabolic parameters in this experimental model. These differences cannot be attributed to differences in the affinities for THRs as 3,3′-T2 acts as a more effective and potent THR agonist that binds THRs with higher affinity (98).

## TH metabolites: 3-iodothyronamine (T1AM)

The biogenic amine 3-iodothyronamine (T1AM) is another noteworthy endogenous TH metabolite that has recently been demonstrated to have profound effects on glucose and lipid metabolism at physiological concentrations. Interestingly, these effects were, at least in part, mediated by its actions on hepatocytes.

Tandem mass spectrometry coupled with HPLC allowed to detect T1AM in the blood and in several tissues from both rodents and humans. Tissue concentration of this amine was found to range from 0.2 to 0.3 nM in the blood, to 1–90 nM in other tissues. Although literature is not concordant on the absolute concentration of T1AM in blood and tissue, it is widely accepted that this amine concentrates in some organs, such as liver, brain, and muscle (99, 100). Differently from T3 and its synthetic analogs, T1AM does not bind to the THR, consequently does not share with TH the classical cardiovascular side effects. Most of the effects of this amine is thought to be mediated by the trace amine-associated receptor 1 (TAAR1), a G protein-coupled membrane receptor (46, 101). T1AM can also interact, albeit at lower affinity, with α2A adrenergic receptors and more additional targets, such as apolipoprotein B100, mitochondrial ATP synthase, and membrane monoamine transporters. The physiologic importance of these low affinity interactions is still a matter of investigation. Despite the growing interest for T1AM, the biosynthetic pathway responsible for its production remains quite unclear. The similarity of this amine with T3 allows to speculate that T1AM may be synthesized from this hormone through decarboxylation and deiodination (102–104). Nonetheless, it is unclear where these reactions really occur. In healthy subjects there is a significant correlation between T3 and T1AM serum concentrations (105), suggesting a possible role of the thyroid gland in T1AM synthesis; however, the evidence that thyroidectomized patients treated with thyroxine have normal serum T1AM values, supports the alternative hypothesis that T1AM may be the result of an extrathyroidal metabolism of thyroid hormone (106). Rat peripheral tissues display intracellular T1AM concentration ranging from 5.6 ± 1.5 pmol/g in lung to 92.9 ± 28.5 pmol/g in liver: These concentrations are much higher than those measured in serum (up to 20-fold that measured in the serum (0.3 ± 0.03 pmol/ml) (99). Unfortunately, these results do not allow to clarify if the high tissue concentrations of T1AM resulted from (i) the ability of cell to concentrate it, (ii) intracellular biotransformation of TH, or, (iii) local biogenesis. Experiments performed in H9c2 cardiomyocytes and isolated and perfused rat heart, indicate that intracellular T1AM originate from an active Na^+^ dependent cellular uptake of the amine, and, to a lower extent, from intracellular biotransformation of T3 (99). *In vivo* experiments, using hypothyroid mice treated with an isotope-labeled T4 [heavy-T4 (H-T4)] that can be distinguished from endogenous T4 by mass spectrometry, indicate that T1AM is not an extrathyroidal metabolite of T4, but it is produced by an independent biosynthetic process. Despite these results, the site of T1AM synthesis remains elusive. Nonetheless, the wide organ distribution and relative high concentrations of T1AM, clearly points to a physiological role of this amine. The administration of supra physiological concentrations of T1AM, in rodents, lead to hypothermia, decreased cardiac contractility, behavioral alterations, weight loss, activation of energetic metabolism from lipids, increased gluconeogenesis and impaired insulin secretion.

## T1AM in NAFLD

The effects of T1AM on body weight and energetic metabolism prompted the interest for this amine in pathological conditions characterized by obesity and triglyceride accumulation within the liver. These conditions are often associated to patients with NAFLD, a condition for which there is no effective pharmacological treatment (107). Early findings indicated that central nervous system could be the principal responsible for the acute alterations in glucoregulatory hormones and glucose metabolism seen after T1AM administration to adult male rats (108). On the other hand, the finding that T1AM reaches the higher concentrations in the liver supports the hypothesis that its metabolic effects might be partly mediated by hepatocytes. T1AM uptake, as well its effects on hepatic glucose and ketone body production, has been investigated in perfused liver from healthy rats and in HepG2 r cancer cells (109). When HepG2 cells were exposed to a T1AM (up to 1.0 μM), they concentrated the amine by 6- to 8-fold. Ex vivo experiments with perfused liver confirmed a significant T1AM uptake. In both conditions, T1AM administration produced a significant increase in glucose production, whereas a significant amount of ketone bodies was seen in the liver but not in HepG2 cells known for their limited ketogenic capability (110). This effect may be dependent on pyruvate and amino acid catabolism, so that pyruvate shift toward gluconeogenesis would cause less acetyl-CoA to be available for ketogenesis.

These findings are consistent with a shift of pyruvate toward gluconeogenesis and direct stimulation of fatty acid catabolism occurring in normal hepatocytes (109). Recent investigations confirmed that chronic T1AM administration induces transcriptional changes in hepatocytes and adipocytes consistent with a more lipolytic metabolic pattern (111). Since these effects were confirmed by *in vivo* experiments and were associated to a significant weight loss without changes in food consumption (112), T1AM, or synthetic analogs should be seriously considered as novel therapeutic agents for obese patients with associated NAFLD.

## T1AM and liver cancer

Differently from metabolism-related diseases, much less is known about the effects of T1AM on liver carcinogenesis and neoplastic hepatocytes. Only few papers have investigated the effect of T1AM on cancer cells (113, 114). In a recent paper it was found that micromolar doses of T1AM and its structural analog SG-2, are able to reduce viability and growth rate of the liver cancer cell line HepG2 and the breast cell line MCF7 (114) The exact mechanism by which T1AM altered cell viability and growth rate of these cancer cells is not entirely clear. However, the mitochondrial localization of T1AM suggest a role in metabolic disruption of cancer cell function. The gene expression study suggests that the mechanism of action of T1AM possibly involves its ability to induce upregulation of the expression of the mitochondrial genes *SIRT 4 and SIRT 5*. The upregulation of other genes, such as *G6PD, p53, HIF1a*, and *SIRT 1*, could be indirectly mediated by the metabolic effects of T1AM.

## T1AM and treatment of liver disease

**Figure 1:**
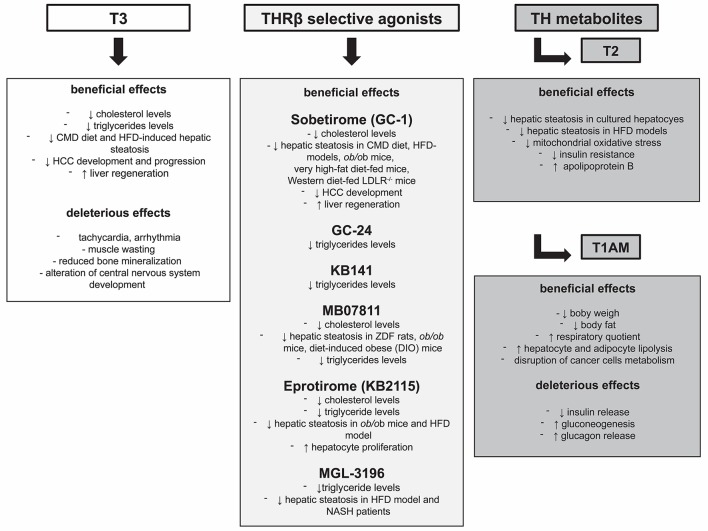
Beneficial and deleterious effects exerted by T3, THRβ selective agonists, and TH metabolites.

Similar to T3, also T1AM modulates lipid and glucose metabolism through central and peripheral actions, as demonstrated by its ability to modulate insulin resistance and hypothalamus-pancreas-thyroid axes in mice after intracranial administration (115). More experimental data are needed to better dissect these two mechanisms of action. Available data are still insufficient to establish a direct role of T1AM and its possible use in the treatment of liver cancer, as further investigations in *in vivo* models are needed. Nonetheless, the possibility that T1AM treatment may result in a reduced risk of liver cirrhosis and HCC development, as a consequence of its potential effect on NAFLD progression should be intensely pursued. A short summary of both beneficial and deleterious effects exerted by T3, THRb selective agonists, and TH metabolites has been reported in Figure 1.

## Conclusion

Liver disease represents an increasing health problem in many countries. From a clinical point of view, a treatment resulting in induction of fatty acid oxidation, decrease in serum triglyceride and cholesterol levels, reduced hepatic steatosis and body weight, without deleterious effects on the heart, might represent a very attractive therapeutic option for many patients. TH, their analogs and metabolites share several desired effects on glucose and lipid metabolism; thus a therapeutic strategy based on their use, as single agents or in association, might be effective for the treatment of liver diseases, such as NAFLD and HCC, that are currently devoid of any effective drug.

## Author contributions

MAK: literature revision and drafting of the article. AP: drafting of the article and figure preparation. AC: coordination of all the work, critical revision of the manuscript and final approval.

### Conflict of interest statement

The authors declare that the research was conducted in the absence of any commercial or financial relationships that could be construed as a potential conflict of interest.

## Abbreviations

HCC: hepatocellular carcinoma
NAFLD: non-alcoholic fatty liver disease
NASH: non-alcoholic steatohepatitis
PH: partial hepatectomy
R-H: Resistant-Hepatocyte
T3: 3,5,3′-triiodo-L-thyronine
THR: Thyroid hormone receptor.
